# Impact of small boat sound on the listening space of *Pempheris adspersa*, *Forsterygion lapillum*, *Alpheus richardsoni* and *Ovalipes catharus*

**DOI:** 10.1038/s41598-023-33684-0

**Published:** 2023-04-28

**Authors:** Louise Wilson, Rochelle Constantine, Matthew K. Pine, Adrian Farcas, Craig A. Radford

**Affiliations:** 1 Leigh Marine Laboratory, Institute of Marine Science, Waipapa Taumata Rau The University of Auckland, 160 Goat Island Road, Leigh, 0985 New Zealand; 2 School of Biological Sciences, Waipapa Taumata Rau The University of Auckland, Private Bag 92019, Auckland, 1142 New Zealand; 3 Department of Biology, University of Victoria, Victoria, BC Canada; 4 Centre for Environment, Fisheries & Aquaculture Science (CEFAS), Lowestoft, Suffolk, UK

**Keywords:** Marine biology, Conservation biology

## Abstract

Anthropogenic stressors, such as plastics and fishing, are putting coastal habitats under immense pressure. However, sound pollution from small boats has received little attention given the importance of sound in the various life history strategies of many marine animals. By combining passive acoustic monitoring, propagation modelling, and hearing threshold data, the impact of small-boat sound on the listening spaces of four coastal species was determined. Listening space reductions (LSR) were greater for fishes compared to crustaceans, for which LSR varied by day and night, due to their greater hearing abilities. Listening space also varied by sound modality for the two fish species, highlighting the importance of considering both sound pressure and particle motion. The theoretical results demonstrate that boat sound hinders the ability of fishes to perceive acoustic cues, advocating for future field-based research on acoustic cues, and highlighting the need for effective mitigation and management of small-boat sound within coastal areas worldwide.

## Introduction

Coastal habitats are diverse environments which supply many ecosystem services including carbon sequestration, climate regulation, erosion prevention, and tourism^1^. These areas also serve as spawning grounds, nursery habitats, and migration stopovers for many species^2^. The high prevalence of human settlements on or near the coastline worldwide means that these habitats are routinely exposed to anthropogenic pressures^3^. In addition to the direct and indirect effects of fishing^4^, impacts also arise from surrounding land use, and can have variable effects depending on the life history stage of species^2^. Research and legislation on the effects of sound pollution (anthropophony) from commercial sources is burgeoning^5–7^, but few environmental impact assessments of human pressures on coastal zones have considered sound from small boats, a highly prevalent stressor^8,9^, which can induce numerous behavioural and physiological effects^10^. Successful conservation and management of coastal habitats requires developing knowledge on how this anthropophony affects different species which produce and/or use acoustic cues.

Communication masking is a widespread effect of anthropophony for marine mammals^11^, which can affect a range of behaviours, depending on the distance of the animal from the source. The same is likely true for vocalising fishes and invertebrates^12,13^. Masking occurs when animals are no longer able to send or receive acoustic cues due to the presence of other sounds (the masker) which overlap in frequency (energetic masking), or are similar to (informational masking), acoustic cues of interest^14^. For fishes and invertebrates, such ecologically important cues include: reef sound, used by larval fishes and invertebrates to navigate towards coastal settlement habitat^15^; the sound of conspecifics, integral for reproduction and competition^16,17^; and the sound of predators, necessary for triggering anti-predation behaviour^18,19^. Therefore, masking of acoustic cues has clear implications for individual fitness and survival.

Communication (or active) space and listening space are two metrics used to assess masking effects caused by anthropophony^20^. These can be used to indicate the ability of animals to send and receive cues of conspecifics (communication space)^21^, or receive cues of con- and heterospecifics, as well as any environmental sounds (listening space)^22^, in the presence of a masker. Since the soundscape of shallow coastal habitats can be highly variable and localised^23^, and there are many sound sources which could provide important sensory cues (not just those of conspecifics), listening space is a useful metric for understanding how sound pollution impacts the ability of coastal taxa to perceive acoustic cues. However, few studies have considered communication masking in fish and invertebrate species, which form the bulk of coastal biomass. Unlike marine mammals, most of these species lack the mobility to move large distances to evade stress. Additionally, most studies^24–27^ documenting anthropogenic impacts upon marine soundscapes have reported sound pressure levels (SPL), but not particle motion. Some fishes have ancillary hearing structures (such as swim bladders) which enable them to sense sound pressure, but most species primarily detect the particle motion component of the sound field^28^, as with invertebrates which are unable to sense sound pressure^12,29^. Since, in the near-field (within 1–2 wavelengths) of a sound source, pressure and particle motion are not directly proportional^30,31^, research seeking to assess masking effects on these species should account for both modalities.

This study investigated the effect of small boat sound on the particle acceleration and pressure (fishes only) listening space of four coastal species common in the Hauraki Gulf, New Zealand: bigeye (*Pemphersis adspersa*), common triplefin (*Forsterygion lapillum*), New Zealand (NZ) paddle crab (*Ovalipes catharus*), and snapping shrimp (*Alpheus richardsoni*). Three of these species produce sound: the bigeye is a nocturnal fish species which produces low frequency ‘pop’ sounds to maintain contact between conspecifics^32,33^; the NZ paddle crab produces three sound types (‘zip’, ‘bass’, and ‘rasp’) associated with breeding and feeding^17^; and the high frequency sound of snapping shrimp is a dominant feature of temperate and tropical marine soundscapes, serving a range of functions including territory defense and mate selection^34,35^. Whilst common triplefins are not known to vocalize, the larvae of this reef species use reef sound as a directional cue^36^. Therefore, sound is believed to be an important sensory cue for all species studied here. By accounting for diel variability in the soundscape, boat speed, and boat proximity, the results of this study highlight how boat sound can reduce the ability of these species to perceive acoustic cues necessary for fitness and survival, in terms of both pressure and particle motion.

## Methods

### Data collection

**Figure 1:**
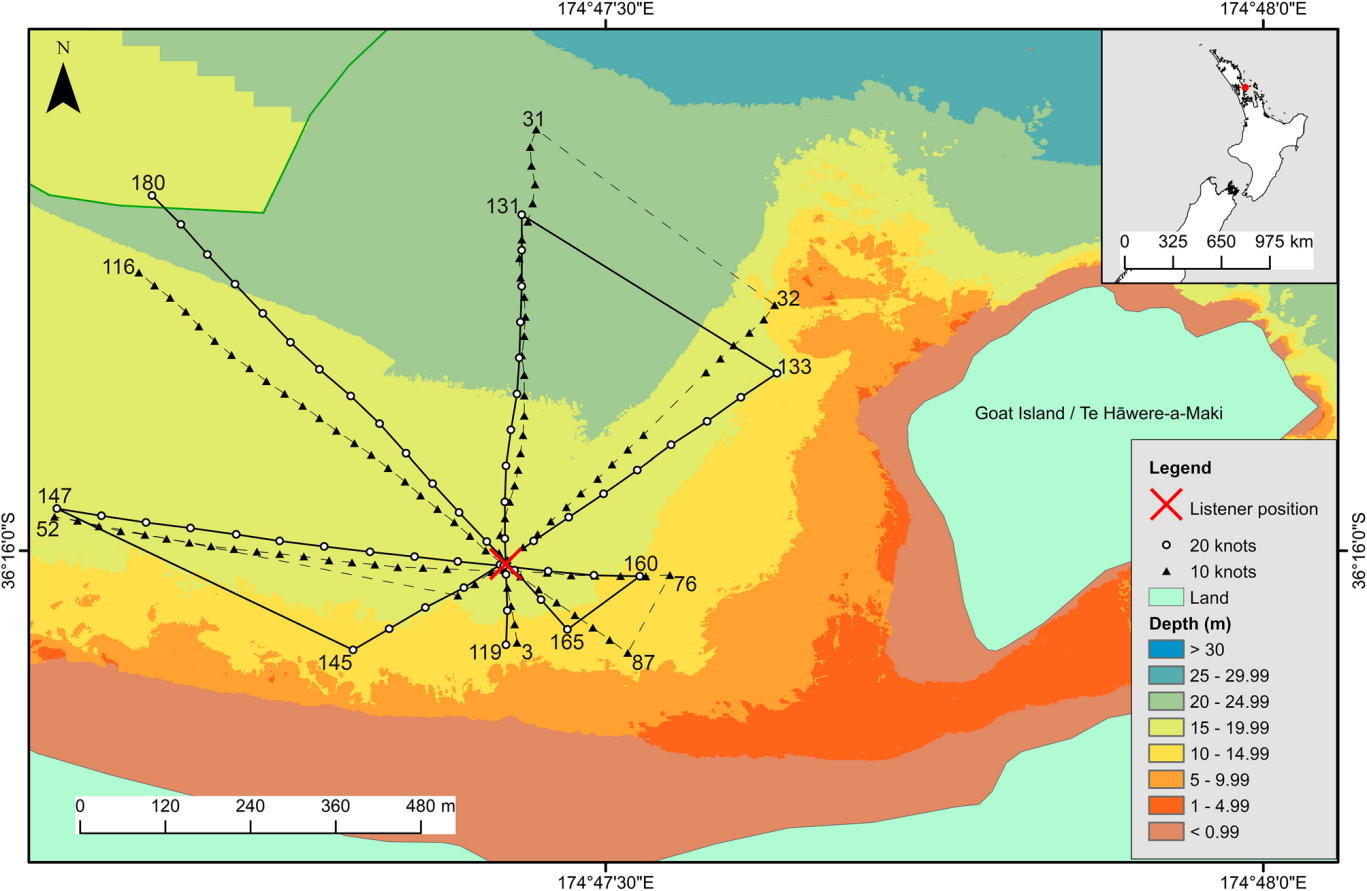
Map of the study site showing transects driven by the small boat at 10 (triangles, dashes) and 20 (circles, line) knots. The division between areas where coarse (20 m) and fine (1 m) resolution bathymetry data were available is illustrated by a green line (top left). The listener position used in Figs. 2 and 3 is marked with a red cross, and numbers at the outer limit of each transect represent the source positions on the x-axis of Figs. 2 and 3. This figure was created in ArcMap (Version 10.8.1; https://support.esri.com/en/products/desktop/arcgis-desktop/arcmap/10-8).

Acoustic data were collected in August 2021 in a sheltered bay within the Cape Rodney-Okakari Point Marine Reserve, a no-take marine protected area (MPA) in Aotearoa New Zealand where recreational and commercial harvesting of all species is illegal (36° 16′ 00.6″ S 174° 47′ 25.5″ E; Fig. 1). This area features a sandy seabed populated with patches of rocky reef and kelp and is a popular site for recreational boating. A hydrophone array was deployed mid-water column (7–8 m depth). The array housed six calibrated hydrophones (HTI-96-MIN; sensitivities −164.8, −164.7, −164.8, −164.9, −165.0, −165.2 dB re 1 V/µPa) orientated equidistant (0.4 m) from each other on three planes (X, Y, Z) (Supplementary Fig. S1). Hydrophones were connected to two four channel recorders (SoundTrap 4300 STD, calibrated by Ocean Instruments NZ) programmed to record continuously (5 min/5 min) at 72 kHz. Mooring weights on the seafloor were used to secure the position of the array, while a sub-surface float maintained the array’s vertical orientation. Guy ropes were used to minimize movement of the array and all loose cables and ropes were secured to prevent sound contamination.

Four transects were driven past the array at 10 and 20 knots (hereafter ‘low speed’ and ‘high speed’, respectively), out to a maximum distance of ~ 600 m (Fig. 1 and Supplementary Fig. S2). The boat used was a 5.8 m aluminum rigid-hulled inflatable boat (RHIB) with a 2.2 m beam and 0.4 m draft, powered by a single Yamaha 150 HP four stroke outboard (total engine power of 111.8 kW). Boats of this length (< 15 m) are common at the study site and in coastal habitats worldwide^37,38^. During the transects, the position of the boat (hereafter ‘source position’) was logged every 5 s using a GPS logger (Holux RCV-3000). No other boats were observed within 2 km of the study site and wind speed (~ 5 knots W – SW) and wave swell (0.2–0.3 m; W – SW) were minimal. A CTD (conductivity, temperature, depth) cast (SonTek CastAway-CTD) was used to obtain a temperature, density, and sound speed profile of the water column.

Bathymetry data (1 m resolution) for the majority of the study site, extending up to ~ 1.2 km from the coastline, were available from a prior study^39^ (Fig. 1). Lower resolution (20 m) bathymetry data were obtained from NIWA for the area which the higher resolution data did not cover^40^.

### Acoustic analyses

Acoustic data were processed in Matlab (2020b). The cut-off frequency (ƒ_c_), the frequency below which sound will not propagate^31^, was calculated at each source position using the following equation^41,42^:

1 $${f}_{c}= \frac{{c}_{w}}{4 D \sqrt{1-{(\frac{{c}_{w}}{{c}_{b}})}^{2}}}$$

where D (m) is water depth, c_w_ is the CTD derived sound speed in seawater (1507 m s^-1^), and c_b_ is the sound speed in sandy sediment (1650 m s^-1^), taken from Jensen et al.^43^. D for each source position was extracted from the available bathymetry data. Any source positions where ƒ_c_ was ≥ 80 Hz were removed from the data set to permit analysis of acoustic data in full octave level bands (FOL base 10) from 125 to 2000 Hz. Similarly, by extracting D at a range of positions across the study site, it was confirmed that the ƒ_c_ of the direct path between the hydrophone array and all remaining source positions was below 80 Hz.

Acoustic data from the two recorders were aligned using the function ‘AlignWave’^44^, using cross-correlation to sync the time-series waveform data from each hydrophone. For this purpose, a 5 s chunk of acoustic data was selected per hydrophone per source position, spanning 2.5 s before and after the time of each source position. Pressure data from all six hydrophones were averaged, then converted to dB to provide a single received SPL (dB re 1µPa) per source position, representative of the sound level in the centre of the hydrophone array. Power spectral density (PSD) analysis was carried out on the mean received SPL (dB re 1µPa) for each source position in 1 Hz bins using a 1 s Hamming window with 50% overlap (Supplementary Fig. S2).

Euler’s equation of motion was used to convert the received SPL (dB re 1µPa) from the paired hydrophones on each plane (X, Y, Z) to acceleration (dB re 1 m s^-2^)^45,46^:

2 $${a}_{x}= \frac{{p}_{2}- {p}_{1}}{\rho d}$$

3 $${a}_{y}= \frac{{p}_{4}- {p}_{3}}{\rho d}$$

4 $${a}_{z}= \frac{{p}_{6}- {p}_{5}}{\rho d}$$

where a = acceleration (m s^-2^), p = pressure (µPa) at each hydrophone, $\rho$ = density of medium (1026 kg m^-3^) and d = distance between hydrophones (0.4 m). The acceleration magnitude (m s^-2^) was then calculated:

5 $$\mathrm{a}=\sqrt{{{a}_{x}}^{2}+{{a}_{y}}^{2}+{{a}_{z}}^{2}}$$

PSD analysis was then carried out on the acceleration magnitude data for each source position using a Hamming window with 50% overlap and window size of 2048 samples (Supplementary Fig. S2).

### Modelling boat source level

Third octave level (TOL) PSD analysis was carried out on the mean acoustic data (RL_*f*_) for each source position using a 1 s Hamming window with 50% overlap. The depth-averaged transmission loss (TL_*f*_) between each pair of boat and recorder positions was calculated for each 1/3 octave band using an energy-flux numerical model based on Weston’s Equations^47^, which accounts for the range-dependent bathymetry and the sediment reflectivity characteristics. The acoustic properties of the seawater and of the seafloor sediment were taken as in the parametrization of Eqs. (1) and (2). The source pressure spectrum for each boat position was then calculated using:

6 $${\text{SL}}_{f} = {\text{RL}}_{f} + {\text{TL}}_{f}$$

where by the source level, *SL*, for frequency, *f,* was the sum of the transmission loss, *TL*, and received level*, RL*, for *f*. The SL at source positions within 50 m of the hydrophone array were more variable, due to simplifications of the transmission loss model and the time-averaging procedure used. Therefore, medians were calculated over all source positions > 50 m from the hydrophone array, per speed, to define the speed-dependent source spectrum. Therefore, the sound source was assumed to be omnidirectional.

### Modelling received sound pressure levels at a range of positions across the study site

The median SL_*f*_ for each boat speed was then used to model the RL_*f*_ at an array of receiver positions around each source position, where an individual animal could potentially be. The depth-averaged TL around each source position was calculated along 72 radials (5° separation), extending out to 3000 m with 1 m spatial resolution using the Weston energy flux model, as detailed previously. The depth-averaged received levels at each 1 m range step were then calculated by subtracting the range-dependent TL_*f*_ from the SL_*f*_ at each receiver position across third octave bands between 125 and 2000 Hz. Any receiver positions where ${f}_{c}$ was greater than 80 Hz were excluded from the dataset.

### Converting received sound pressure levels to acceleration

Received third octave sound pressure levels (TOL SPL, dB re 1µPa) at each receiver position were converted to acceleration (dB re 1 µm s^-2^) using the following equations from Nedelec et al.^42^ and Chapman and Hawkins^48^:

7 $${\delta }_{near-field}= \frac{p}{2\pi f\rho c} {\left[{ 1+\left( \frac{\lambda }{2\pi r}\right)}^{2} \right]}^\frac{1}{2}$$

8 $${\delta }_{far-field}= \frac{p}{2\pi f\rho c}$$

9 $$\lambda =\frac{c}{f}$$

10 $$a= \delta 2\pi {r}^{2}$$

where $\delta$ = displacement (m), p = pressure (Pa), $f$ = frequency (Hz), ρ = density of sea water (kg m^-3^), c = speed of sound in sea water (m s^-1^), $\lambda$ = wavelength (m), r = distance from receiver position to source position (m) and a = acceleration (m s^-2^). The displacement calculation used depends on whether the receiver position is in the near-field or the far-field of the sound source (Eqs. 8 and 9). It was therefore necessary to calculate, for each source position and each $f$, which receiver positions were within $2\lambda$ of the source position^15^.

The auditory bandwidths used by species in this study are unknown, and difficult to measure^11^. Therefore, it was assumed that the critical bandwidths of all species studied here can be approximated by full octave bands, as has previously been applied to study masking effects on fishes^7,20^. Therefore, TOL received levels in pressure and acceleration were converted to FOL.

### Listening space reduction analysis

At each receiver position, the depth-averaged listening space reduction (LSR) during noise exposure from the small boat was calculated for four coastal species, using the methods outlined in Pine et al.^22^, in terms of both sound pressure (dB re 1µPa) and particle acceleration (dB re 1 µm s^-2^):

11 $$LSR \left(\mathrm{\%}\right)=100 \left(1-{10}^{2 \frac{{NL}_{2}- {NL}_{1}}{N}}\right)$$

where, for each FOL, NL_2_ = received sound pressure level from the passing boat (i.e. the masker), NL_1_ = maximum of ambient SPL and hearing threshold level of a particular species (listener), and N = TL coefficient (slope of curve-fitted TL values). A key assumption in the LSR calculation for particle motion is that the energy decay behaves similarly to pressure^48^.

Hearing threshold data (pressure and particle acceleration) for bigeye and common triplefin were obtained from Radford et al.^49,50^. Particle acceleration audiograms for the NZ paddle crab and snapping shrimp were obtained from Radford et al.^29^ and Dinh and Radford^34^, respectively. Hearing threshold data for each species were converted to FOL values by linear interpolation^22^ (Supplementary Fig. S3). Baseline day and night time ambient SPLs (dB re 1µPa) for the study site during August 2020, at times when boats were absent, were obtained from Wilson et al.^8^. To derive these values, PSD analysis was computed on all acoustic files recorded during August 2020 using a 1 s Hamming window with 50% overlap. Following the removal of files where boat sound was detected, the 50th percentile (dB re 1µPa) within each 1 Hz band for all day and all night time recordings was calculated (Supplementary Fig. S3). These values were converted to acceleration using the same protocol as above, assuming all sound sources to be far-field. LSR values were then averaged across all FOL to provide a single LSR score for the bandwidth over which audiogram data were available for each species.

**Figure 2:**
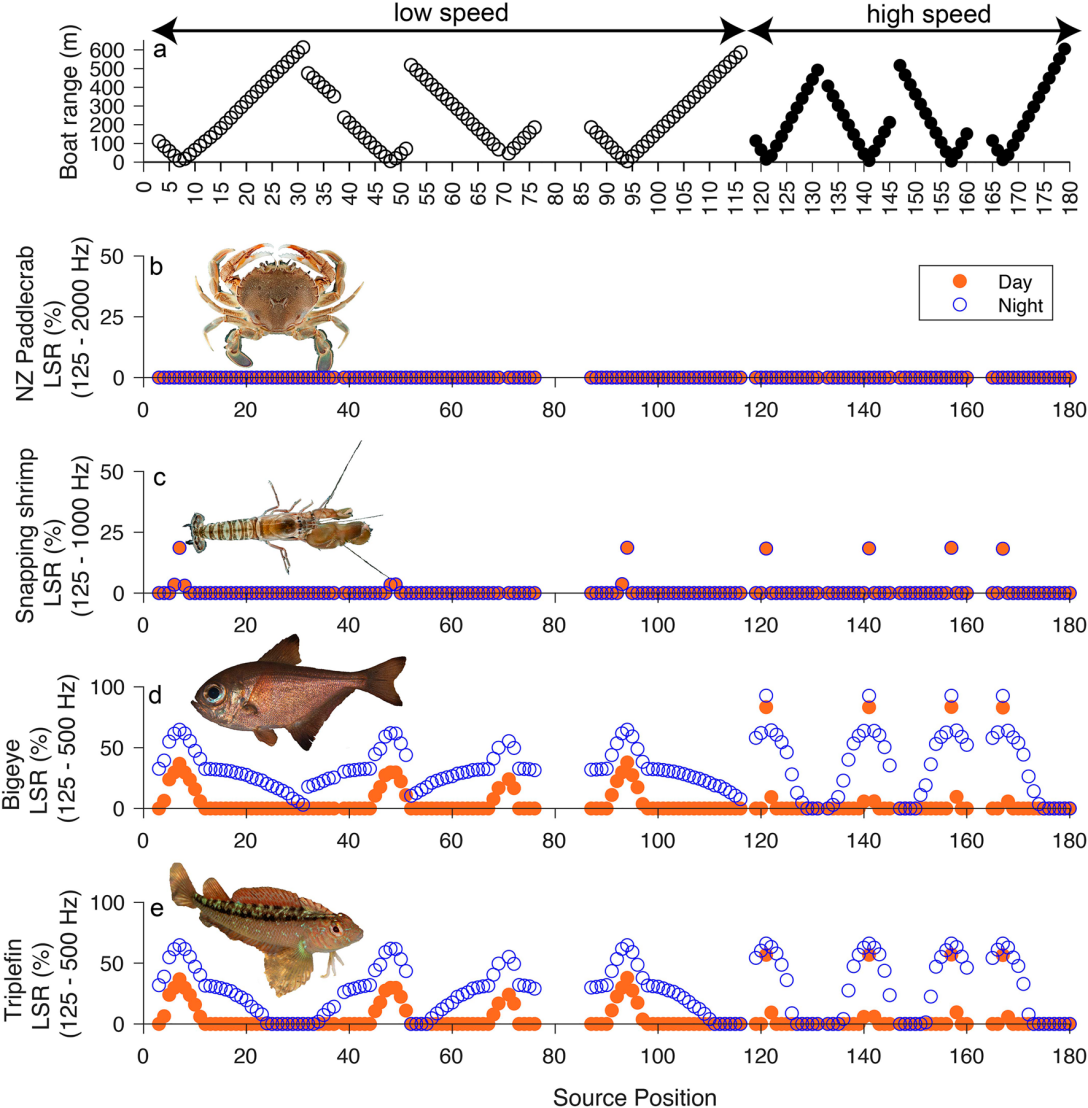
Received particle acceleration level (dB re 1 µm s^-2^) listening space reductions (%) for the four study species when a boat source was present at a range of distances from the listener. Listening space reductions during day (orange, filled circles) and night (blue, hollow circles) time ambient conditions at the listener position are presented. The proximity of the boat to the listener position (where 0 m = listener position) and boat speed (hollow circles = low speed; filled circles = high speed) at each source position are illustrated (**a**). Note the y-axis scale used for fishes and invertebrates is different. (Fish images by Paul Caiger; invertebrate images by Richard Taylor).

**Figure 3:**
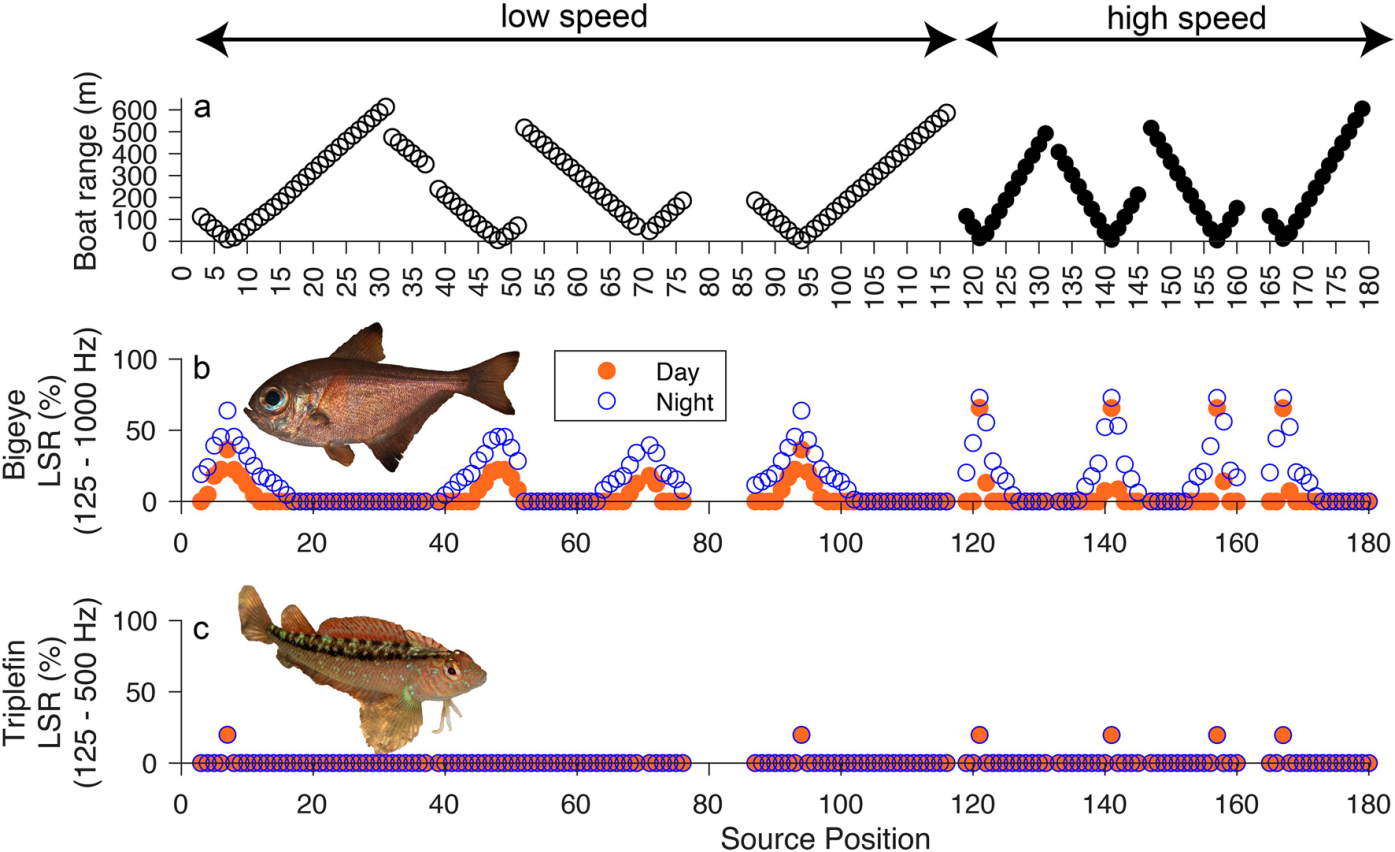
Received sound pressure level (dB re 1µPa) listening space reductions (%) for bigeye and common triplefin when a boat source was present at a range of distances from the listener. Listening space reductions during day (orange, filled circles) and night (blue, hollow circles) time ambient conditions at the listener position are presented. The proximity of the boat to the listener position (where 0 m = listener position) and boat speed (hollow circles = low speed; filled circles = high speed) at each source position are illustrated (**a**). (Fish images by Paul Caiger).

**Figure 4:**
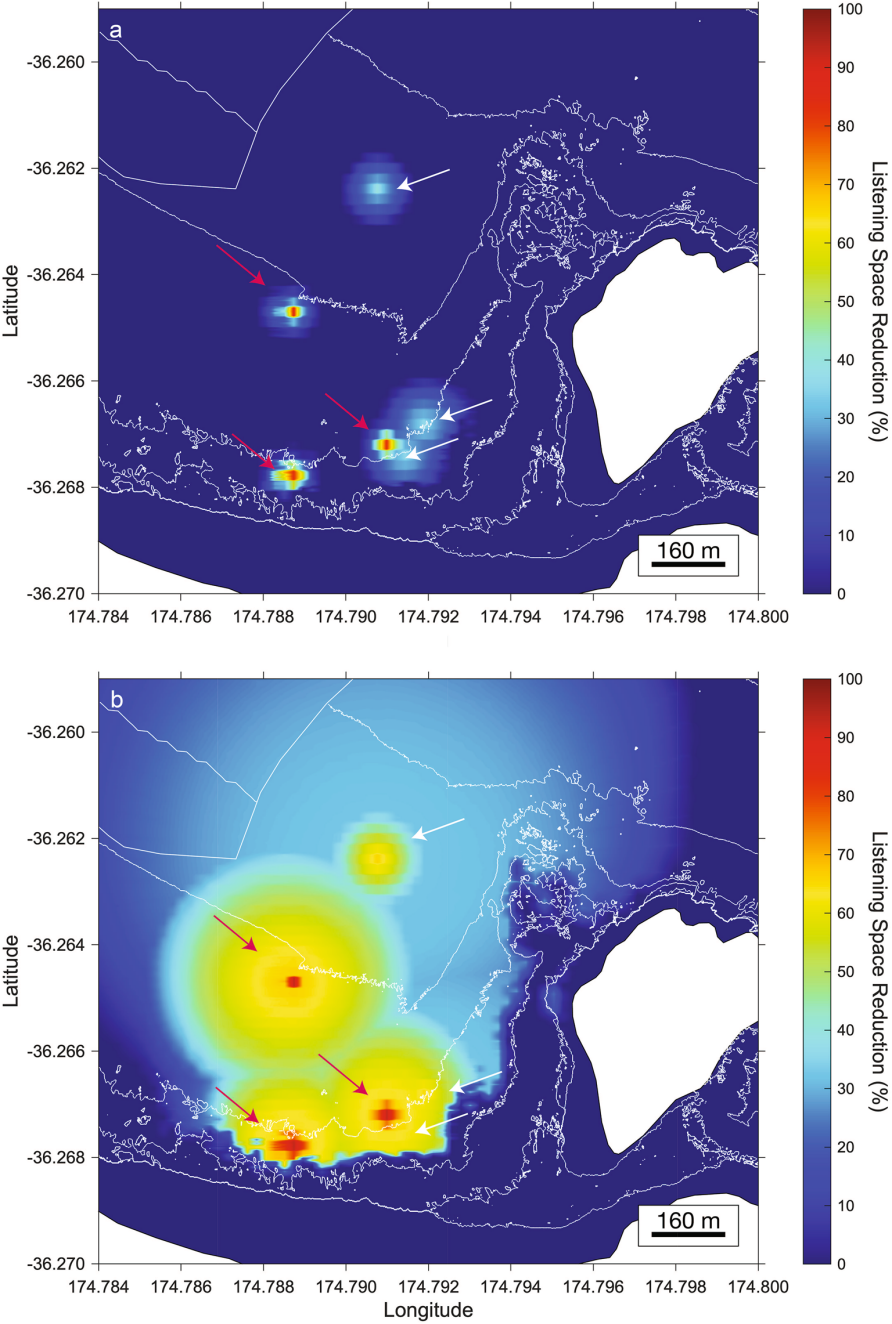
Received particle acceleration (dB re 1 µm s^-2^) listening space reductions (%) for bigeye during the day (**a**) and night (**b**) while a boat is present at six of the source positions modelled in this study. Source positions where the high speed SL was applied are highlighted with a red arrow, those where the low speed SL was used are highlighted with a white arrow. The six source positions were selected randomly using a random number generator. This figure was created in Matlab (Version 2020b; https://matlab.mathworks.com/).

To allow comparisons between species and boat speeds for each sound modality, LSR values at a single receiver position at the centre of all eight transects (hereafter ‘listener position’, red cross in Fig. 1) were calculated for all source positions during day and night time ambient conditions (Figs. 2 and 3). These results highlighted that the impact of boat sound on LSR was greatest for bigeye, a nocturnal species. Received particle acceleration LSR values for bigeye during day and night when a boat was present at six randomly selected source positions were then mapped (Fig. 4), illustrating variability in the impact of boat sound on bigeye occurring across the study area during a period of high boating activity. Linear interpolation was used to smooth between the 216,000 receiver positions surrounding each source position.

## Results

Boat speed, source position (i.e. proximity of the boat to the listener), ambient condition, and sound modality all influenced the listening space available to each species at the listener position (Figs. 2 and 3).

### Impact on listening space of crustaceans

There was no effect of boat sound on the listening space of NZ paddle crab during day or night. For snapping shrimp, listening space was reduced by up to 18.6% when the boat source was within 5–15 m of the listener position (Fig. 2). The impact of boat sound on snapping shrimp listening space was similar irrespective of boat speed and ambient condition, and peak LSR was always at the closest point of approach (CPA). Beyond 35 m, there was no effect of boat sound on the listening space of snapping shrimp.

### Impact on listening space of fishes

In daytime conditions at low speed, particle acceleration LSR was ≥ 30% for bigeye and common triplefin when the source was within 24 m of the listener position, but LSR > 15% occurred up to 74 m (16.7% for both species) from the source (Fig. 2). Peak daytime LSR for bigeye (83.5%) and common triplefin (57.0%) occurred during high speed CPAs when the source was within 5 m of the listener position. Boat sound impacted the listening space of both species across a greater area during night-time conditions, particularly at low speed—up to 613 m from the source for bigeye (3.0% LSR), and up to 431 m (0.5% LSR) for common triplefin. Whilst low speed resulted in LSR across a greater area, high speed resulted in greater LSR at distances close to the source for both species. For example, at low speed, LSR was < 50% beyond 64 m from the source, but at high speed LSR was > 50% within 115 m of the source (Fig. 2d and e). For both species, overall peak LSR occurred under nighttime conditions at high speed–92.78% for bigeye, and 66.1% for triplefin.

These general findings are consistent for bigeye during both day and night at all listener positions surrounding the six randomly selected source positions (Fig. 4). During daytime conditions, the maximum distance that low speed impacted listening space at the three source positions was 105–130 m, but LSR was < 25% beyond 47–64 m from the boat source. For high speed, the maximum distance that boat sound impacted listening space was 76–93 m, with LSR < 25% beyond 51–54 m. At night, the range over which boat sound impacted LSR by over 25% increased to a maximum of 375–484 m at low speed, and 266–323 m at high speed. The maximum distance that boat sound impacted listening space was 860 m from the source (0.4% LSR) at low speed and 420 m from the source (0.6% LSR) at high speed.

### Importance of sound modality

The effect of boat sound on the particle acceleration listening space of both fish species were similar (Fig. 2), but the impact of boat sound on pressure LSR was greater for bigeye at both speeds during daytime and night-time conditions (Fig. 3). At low speed, LSR ranged from 20.6 to 36.5% within 5 – 34 m of the source during the day, and 43.0 – 63.8% at night. At high speed, LSR ranged from 7.2 to 65.7% within 5–39 m of the source during day, and 52.3–72.8% at night. Low speed impacted listening space out to a maximum range of 96 m during the day (5.8% LSR), and 218 m at night (1.3% LSR). At high speed, impacts extended out to 59 m (8.5% LSR) during the day, and 250 m (0.8% LSR) at night. In contrast, only boat sound within 15 m had an effect on the listening space of common triplefin, which was reduced by 19.6 – 19.8%, irrespective of the ambient condition.

## Discussion

This study indicates that the particle motion listening space of fishes living in a coastal MPA is reduced by ≥ 30%, when a small boat approaches within a 24 m radius. In contrast, the effect of boat sound on the listening space of crustaceans was minimal, with no effect at all on NZ paddlecrab. Particle motion LSR at the listener position peaked during high-speed CPAs under night time conditions (Fig. 2), reaching maxima of 92.8% for bigeye, 66.1% for common triplefin, and 18.6% for snapping shrimp. Greater impacts on fishes can be explained by their enhanced hearing sensitivity, and diel variation was driven by quieter ambient levels at night, when soniferous activity of reef species was lower^8^. In terms of sound pressure, the contrasting results for fish species highlight the importance of considering both sound pressure and particle motion when assessing communication masking in fishes^42,51^.

Compared to the fishes studied here, snapping shrimp and NZ paddle crab have poorer hearing abilities (Supplementary Fig. S3). Consequently, the impacts of boat sound on these species is audiogram limited^11^, and there was no difference between the effects of boat sound during day and night on either species. Below 250 Hz, snapping shrimp have a lower hearing threshold than NZ paddle crabs, which was reflected in greater LSR for snapping shrimp across all transects at both speeds (compared to NZ paddle crab). Whilst boat sound only impacted snapping shrimp listening space by > 18% when the boat source was within 15 m of the listener, these findings highlight the need for further field-based research investigating the impact of sustained periods of boating activity in busy coastal habitats, when multiple boats may travel within close proximity of coastal taxa for prolonged durations. During agonistic interactions, snapping shrimp display their chela and use them to generate impulsive snaps by cavitation of air bubbles^52,53^. The inability of competitors to detect these snaps could inhibit the ability of snapping shrimp to defend territories and identify dominant competitors, increasing the time needed to resolve such encounters. Thus, boat sound could impact the range over which individuals are able to hear the cues of conspecifics, with clear consequences for reproduction and competition.

All fishes hear by particle motion, but the presence of gas-filled organs and ancillary structures in some species can broaden the frequency range across which animals can hear by transducing pressure to particle motion^28,54^. This is the case in bigeyes^50^, which likely explains why the impact of boat sound on bigeye listening space is similar regardless of sound modality (Figs. 2 and 3). Common triplefins, however, are a bottom-dwelling species which lack a swim bladder. As a result, boat sound has almost no effect on common triplefin listening space in terms of pressure (Fig. 3), but a similar effect is found for both fish species when considering particle motion (Fig. 2). Thus, whilst the impact of boat sound for the invertebrate species studied is limited by their hearing abilities (audiogram limited), the greater particle motion hearing sensitivity of the fish species means that the effect of boat sound is limited by variability in ambient sound (ambient limited) (Fig. 2)^11^. As such, differences between the effects of boat sound during day and night are evident for both bigeye and common triplefin, with higher LSR at night, due to the night-time soundscape at Goat Island being up to 14 dB re 1µPa quieter than during the day^8^.

Furthermore, Radford et al.^33^ found bigeye active space to be greatly reduced during summer when ambient sound levels were louder than at other times of the year due to increased biological activity. Therefore, communication masking for this species is likely to be greatest in summer when both boat activity and the activity of soniferous animals is highest. Higher LSR at night, coupled with the nocturnal activity of this species^33,55^, suggests that communication masking would be greatest during dusk and night, dusk being a time when many boats are returning to harbor. The quieter night time soundscape at Goat Island may mean that there is more space in the acoustic scene for bigeye to adapt to anthrophony and maintain communication during this time. For example, bigeyes may be able to increase the amplitude of their calls, a strategy adopted by other fish species^56,57^. However, prior research in this region shows that the soundscapes of nearby coastal habitats are louder at night^8^. Thus, these results highlight the importance of considering the ambient soundscape when assessing the impacts of anthropogenic sound on marine species, which varies considerably over short spatial and temporal scales in coastal habitats^58,59^, but is rarely accounted for in communication masking studies.

Juvenile bigeyes feed during the day and are found close to their shelters. Consequently, communication masking is also likely to vary depending on bigeye life history stage. The same is likely true for triplefins, the planktonic larvae (*Forsterygion* spp.) of which use reef sound to direct nocturnal swimming behaviour towards settlement habitat^60^. In contrast, adult triplefins are diurnal, bottom-dwelling species which are highly territorial and sedentary^61^. While males nest guard, females travel from nest to nest in search of the largest males and nests^61^. A reduction in the ability of these species to perceive acoustic cues at multiple stages in their life history could impact reef recruitment, territory defense, and/or shoaling size, impacting fitness and survival, and possibly leading to population and ecosystem scale effects. These impacts are likely to be most pronounced in areas of high boat traffic, such as shallow coastal habitats like the MPA studied here, where listening space is frequently reduced as boats come and go at variable speeds and proximities.

Many fishes and invertebrates are territorial and stay in a localised area during breeding to guard nests or mates (e.g. common triplefin, NZ paddle crab), or display site fidelity to favorable habitat type (e.g. bigeye). However, some fish and invertebrate species are able to increase call rate^62–64^, which may improve the likelihood of being heard by conspecifics when listening space is restored between successive boat passages. Since the calls of fishes and invertebrates display wide inter-individual variation^17,65,66^, the ability to alter call rate is likely to be constrained by body size and condition^67^. Another coping strategy is to switch to predominantly visual displays^68^, a strategy used by some terrestrial species living in loud habitats^69^. Other species have been found to: reduce call rate in the presence of boat sound, possibly to avoid unnecessary energy expenditure^70,71^; move away to quieter areas^45,72^; and spend less time feeding^73^. Such masking release mechanisms may incur energetic and fitness costs as well as increased risk of predation^62,67^. For example, adjusting call rate may impact successful and efficient reproduction since females of some species rely on temporal variation of male calls to select the fittest males^74^. Also, whilst many of the aforementioned tactics may facilitate communication and/or continued access to food and habitat in the presence of anthropophony, animals may continue to be exposed to physiological stress^75^. In addition, the larvae and eggs of these species are subject to currents, and are unable to move away from acoustic stress.

Accounting for such behavioural responses to anthropogenic sound in the field is challenging, particularly over the spatial scales presented here, and may require tagging of animals^76^, which often requires a permit, or expensive equipment, such as sonar^77^. However, such applications to monitor behavioural responses to boat sound are fairly unexplored^12^. Thus, modelling studies such as these can help to determine the range over which animals may be impacted by boat sound, and guide research questions of focus. Also, for all four species studied here, the results were derived from audiograms calculated using the auditory evoked potential (AEP) method. Since this method does not capture the entire hearing pathway, behavioural methods of measuring hearing are strongly preferred. However, there are very few species for which particle motion behavioural audiograms are available, and this should be addressed in future research. The measurement of particle motion source levels of the vocalisations of different species would also greatly benefit this field. Additionally, future work should seek to directly measure particle motion at a range of locations across habitats of interest, using a vector sensor or accelerometer. Whilst such calculations would be substantially limited in area due to time and equipment constraints, such data would help to validate propagation model based studies such as those presented here, and would allow listening space calculations to be made anywhere in the study area, regardless of depth. Since small boats vary widely in their source level, and propagation conditions in coastal habitats are complex, the repetition of this work using different/multiple boats at different study sites would also be advantageous.

In summary, the effect of boat sound on coastal species results from the complex interplay of a species’ ecology, local soundscape conditions, and boating activity. Existing knowledge on the effects of anthropophony on fishes and invertebrates has largely been limited to lab studies^12^, where propagation conditions vary substantially from those experienced in the field, and few studies have considered impacts on communication using particle motion. This study furthers knowledge of how boat sound impacts the ability of coastal species to perceive acoustic cues, by using passive acoustic data to model available listening space in both pressure and particle motion space. The results show that the effects of boat sound on each species are largely dictated by hearing ability and modality, in addition to boat speed and diel fluctuations in ambient sound levels. The impact of boat sound on listening space is also likely to vary throughout the year due to spatio-temporal variation in the soundscape, and changes in the reliance of animals on acoustic cues due to seasonal behaviours such as breeding. Boat sound is one of many stressors which is expected to increase as the global human population, which is denser in coastal regions, continues to rise. The findings presented here have clear consequences for how coastal areas can be managed to reduce acoustic impacts on animals which rely on sound for vital life history functions. In particular, restricting recreational activity is likely to be instrumental in protecting vulnerable coastal habitats. Developments in technology which reduce the acoustic emissions of boats may also catalyze the restoration of coastal soundscapes.

## Supplementary Information

Supplementary Information.

## Data Availability

All code and data used to generate the results and figures presented in this manuscript have been made publicly and freely available online via the University of Auckland’s institutional Figshare: 10.17608/k6.auckland.c.6203761^78^.
