# Supplementary material for: Impact of small boat sound on the listening space of Pempheris adspersa, Forsterygion lapillum, Alpheus richardsoni and Ovalipes catharus
Source: Sci Rep. 2023 Apr 28;13:7007. doi: 10.1038/s41598-023-33684-0 (PMC10147705; doi:10.1038/s41598-023-33684-0)
Supplement: Supplementary file 1 — Supplementary Information. [file 41598_2023_33684_MOESM1_ESM.docx]

**Title**

Impact of small boat sound on the listening space of *Pempheris adspersa*, *Forsterygion lapillum*, *Alpheus richardsoni* and *Ovalipes catharus*

**Author names and affiliations**

Louise Wilson* ^a^, Rochelle Constantine ^a,b^, Matthew K. Pine ^c^, Adrian Farcas ^d^, Craig A. Radford ^a^

^a^ Leigh Marine Laboratory, Institute of Marine Science, Waipapa Taumata Rau The University of Auckland, 160 Goat Island Road, Leigh, 0985, Aotearoa New Zealand

^b^School of Biological Sciences, Waipapa Taumata Rau The University of Auckland, Private Bag 92019, [Tāmaki Makaurau](https://www.aucklandcouncil.govt.nz/arts-culture-heritage/heritage/Pages/tamaki-makaurau-aucklands-heritage.aspx) Auckland 1142, Aotearoa New Zealand

^c^ Department of Biology, University of Victoria, British Columbia, Canada

^d^ Centre for Environment, Fisheries & Aquaculture Science (CEFAS), Lowestoft, Suffolk, UK

**Corresponding author**

Louise Wilson, +44 7934 189736, lwil634@aucklanduni.ac.nz


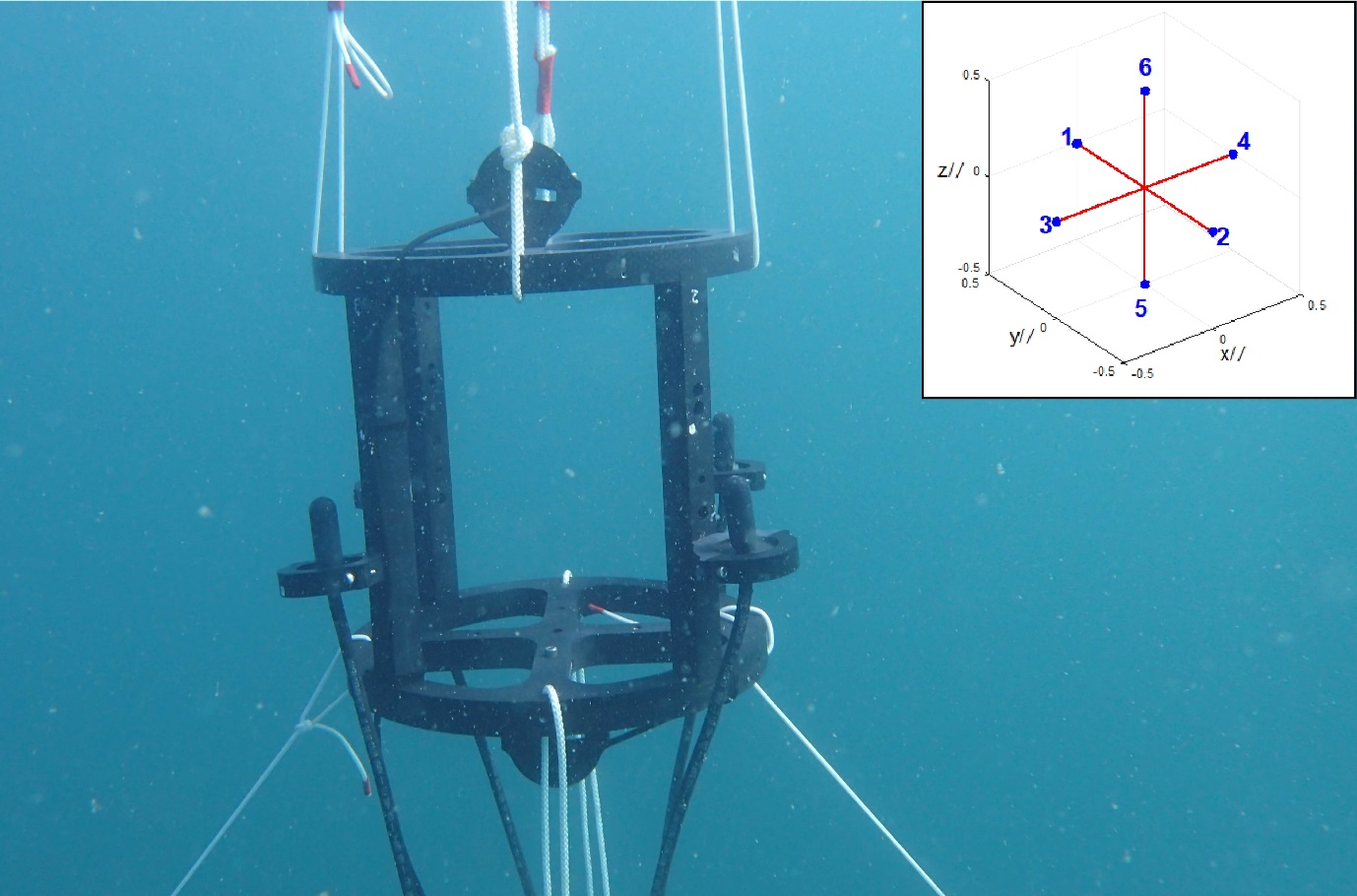


Supplementary Figure S1. Hydrophone array used to collect acoustic data in the present study. The inset image (top right) illustrates the orientation of the six hydrophones on the X, Y, and Z planes. The array is constructed of acoustically transparent material. (Main image supplied by LW, inset image provided by Peter Rogers)


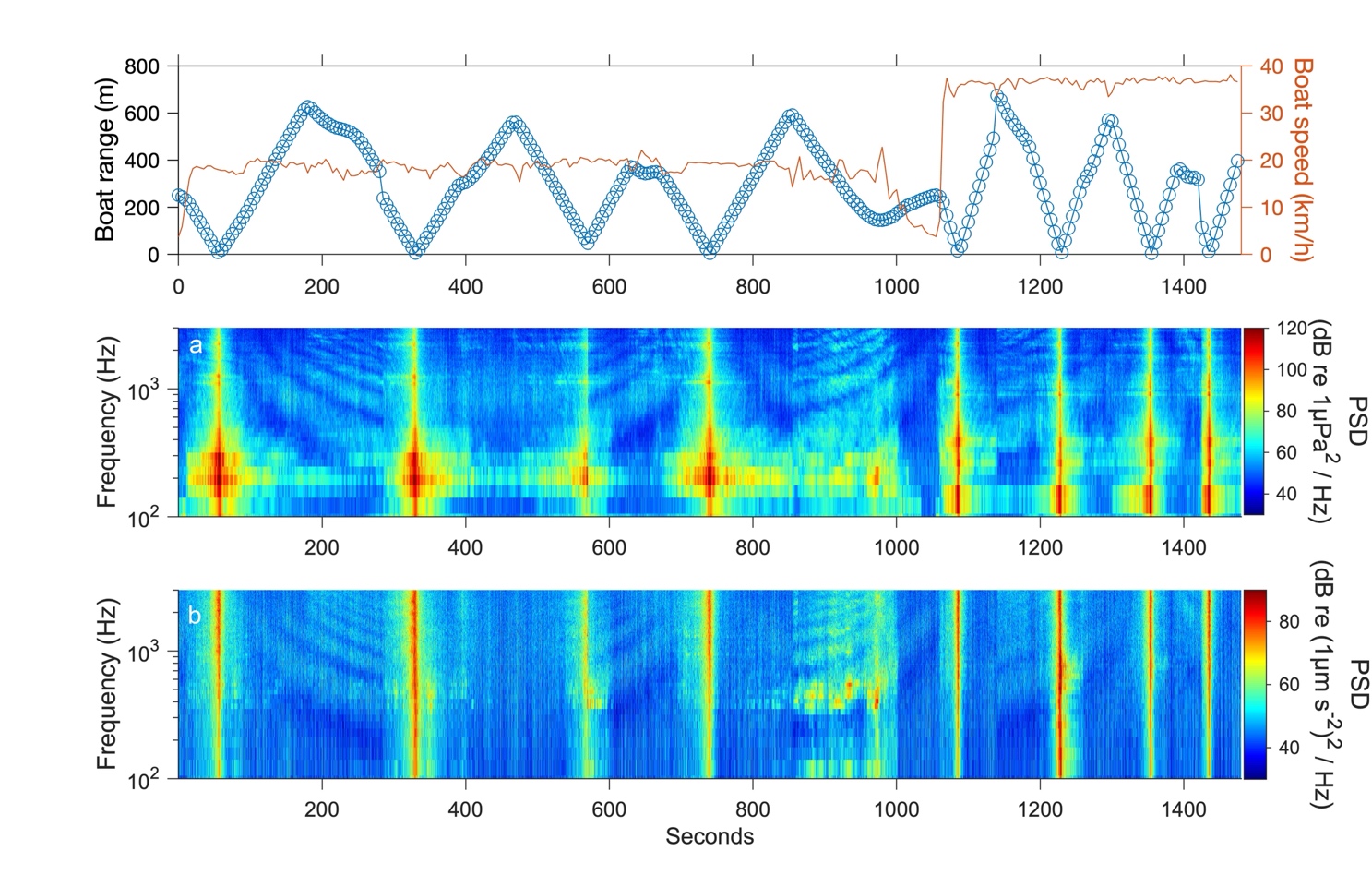


Supplementary Figure S2. Power spectral density of (a) received sound pressure levels (dB re 1µPa^2^ / Hz) and (b) received magnitude (dB re 1 (µm s^-2^)^2^ / Hz) during boat transects. The boat was travelling at ~ 10 knots for the first four approaches, and ~ 20 knots for the second four approaches.


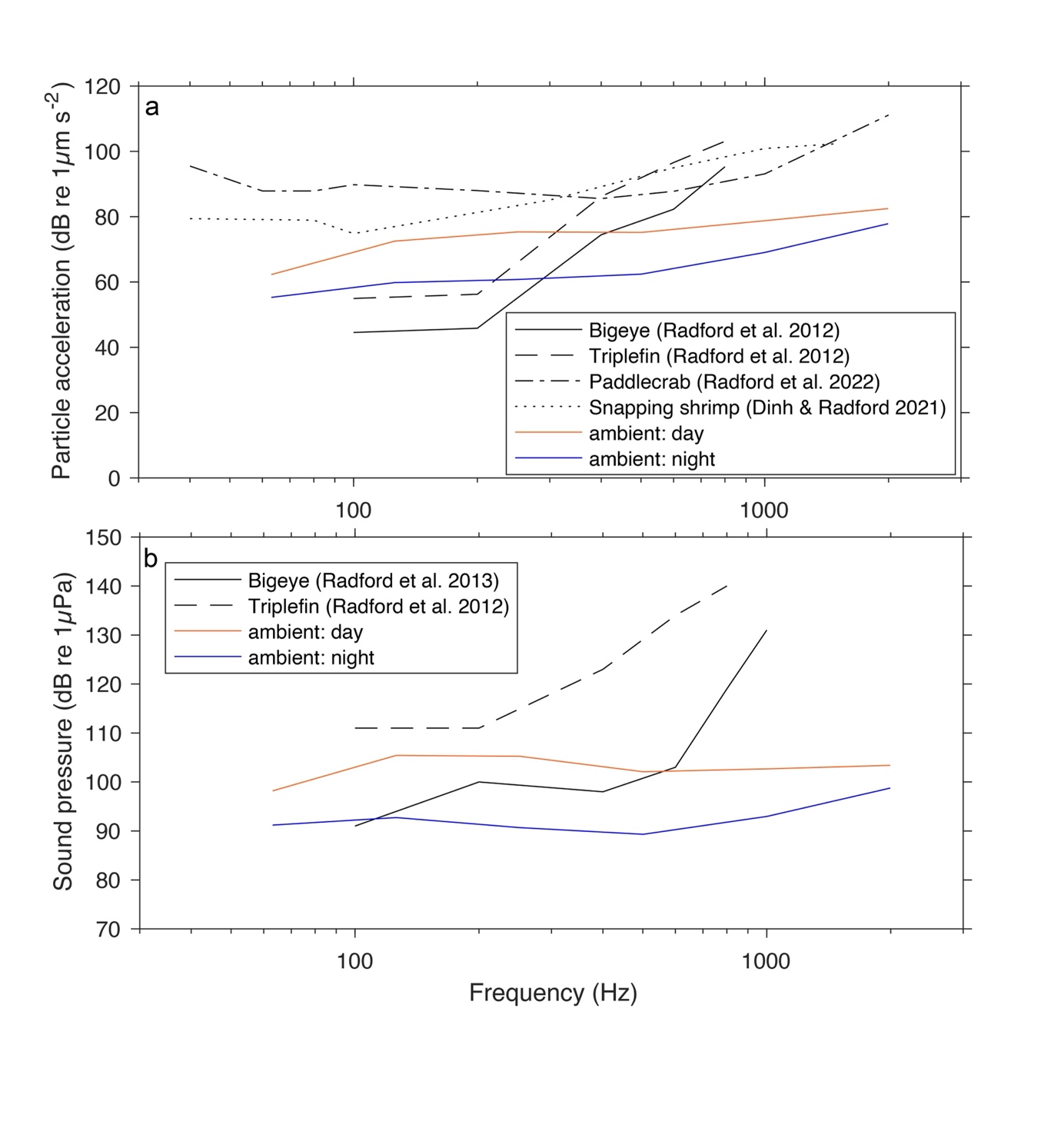


Supplementary Figure S3. (a) Particle acceleration (dB re 1µm s^-2^) and (b) sound pressure (dB re 1 µPa) hearing thresholds for the four species studied in this experiment. Day- and night-time ambient levels (50^th^ percentile) recorded at the study site during August 2020 are also presented.
